# Environmental and diagenetic controls on the morphology and calcification of the Ediacaran metazoan *Cloudina*

**DOI:** 10.1038/s41598-021-90768-5

**Published:** 2021-06-11

**Authors:** Amy Shore, Rachel Wood

**Affiliations:** School of GeoSciences, University of Edinburgh, James Hutton Road, Edinburgh, EH9 3FE UK

**Keywords:** Palaeontology, Evolution

## Abstract

*Cloudina* is a globally distributed Ediacaran metazoan, with a tubular, funnel-in-funnel form built of thin laminae (ca. 1–10 μm). To what degree local environmental controlled morphology, and whether early diagenesis controlled the degree of calcification of *Cloudina,* is debated. Here we test these hypotheses by considering assemblages from four, coeval localities from the Upper Omkyk Member, Nama Group, Namibia, from inner ramp to mid-ramp reef across the Zaris Subbasin. We show that sinuosity of the *Cloudina* tube is variable between sites, as is the relative thickness of the tube wall, suggesting these features were environmentally controlled. Walls are thickest in high-energy reef settings, and thinnest in the low-energy, inner ramp. While local diagenesis controls preservation, all diagenetic expressions are consistent with the presence of weakly calcified, organic-rich laminae, and lamina thicknesses are broadly constant. Finally, internal ‘cements’ within *Cloudina* are found in all sites, and pre-date skeletal breakage, transport, as well as syn-sedimentary botryoidal cement precipitation. Best preservation shows these to be formed by fine, pseudomorphed aragonitic acicular crystals. Sr concentrations and Mg/Ca show no statistically significant differences between internal *Cloudina* cements and botryoidal cements, but we infer all internal cements to have precipitated when *Cloudina* was still *in-situ* and added considerable mechanical strength, but may have formed post-mortem or in abandoned parts of the skeleton.

## Introduction

The terminal Ediacaran saw the appearance of evolutionary innovations such as the first appearance of supposed metazoans with motile behaviour and biomineralised skeletons^1,2^. *Cloudina,* and cloudinomorphs (or cloudinids)—a term defining a group of similar tubular fossils including *Conotubus, Saarina, Multiconotubus, Costatubus, Zuunia,* and *Rajatubulus*—were globally distributed between ca. 550-ca. 522 million years ago (Ma)^1,3,4^. Cloudinomorphs share a similar, funnel-in-funnel organisation without transverse structures, with a straight or sinuous morphology^1,5^. They can be organic-only or preserved as calcite, phosphate, limonite/pyrite, or silica. Both organic and calcified skeletons may be phosphatised.

Cloudinomorph affinity is unresolved. Recent findings of a pyritised central tubular structure within cloudinomorph tubes from Nevada, inferred to be a gut which, together with the nested-funnel morphology and laminar ultrastructure, suggest an annelid affinity. However, the presence of deep-seated branching within the parent *Cloudina* tube as well as polytomous branching in cloudinomorphs, might indicate a cnidarian affinity^5–7^. Cloudinomorphs may in fact represent a group of diverse taxa with a convergent morphology^7^.

*Cloudina* is a generalist taxa found in multiple carbonate settings, including attached to microbial mats and thrombolites, and capable of reef-framework formation^5,8–10^. Tube size varies inter-specifically and is also environmentally-controlled. For example, in the Nama Group, Namibia, *Cloudina* which grew associated with shallow, hydrodynamically-energetic reefs shows the largest tube diameters recorded, but individuals with smaller tube diameters were dominant in low-energy, microbial mat settings^10^.

*Cloudina* is considered to have either aragonite or high-Mg calcite original mineralogy^1,3,11,12^*,* and is thought to have biomineralised during life. *Cloudina* may have biomineralised via calcification of pre-existing organic laminae as revealed by Raman spectroscopy, which are 1–10 μm in thickness. These are often paired, with up to eight laminae within a wall^14,15^. The walls of *Cloudina* show a granular, micritic microstructure (crystal size ca.1 μm), even in phosphatised specimens^13^. This calcification may have proceeded via growth of initial amorphous calcium carbonate (ACC) nanoparticles, comparable to that found in modern echinoderms, molluscs and cnidarians^16^.

*Cloudina* are observed to show both brittle fracturing of the tube walls, suggesting early, probable in-vivo and potentially strong calcification, as well as ductile deformation suggesting weaker calcification of organic-rich laminae^3,13^. It has been argued that delamination structures (where laminae peel apart from one another) in *Zuunia*, and widespread plastic deformation in cloudinomorphs generally, suggests a skeleton of primarily organic composition, where calcification was post-mortem and diagenetically-mediated^15^. Large sparry calcite crystals that incorporate multiple laminae and infill *Cloudina* tubes from the Mooifontein Member in the Witputs Basin of the Nama Group, have been noted, which has been used to further support the notion that *Cloudina* tube walls consist of diagenetic, rather than biotic, calcite^15^. But it has long been known that acicular, pseudomorphed aragonitic cements are present between the laminae of *Cloudina*, which are often neomorphosed to a fibrous or sparry calcite^3^, and these areas have been found to be organic-rich in specimens from Brazil^14^.

Identifying biologically controlled vs. biologically induced calcification^17^ can be problematic, as the degree of biological control can vary. Indeed, limited or considerable biological control can still be demonstrated even where most aspects of calcification vary with environmental parameters^18,19^. Conversely, apparently organised microstructures may not result from biological control per se but may reflect interactions among otherwise disorganised crystals^20^.

Here we test the hypothesis that the type and extent of calcification and morphology in *Cloudina* was controlled environmentally, by considering coeval assemblages from the Upper Omkyk Member of the Nama Group, Namibia, from diverse water depth and hydrodynamic settings along an inner shelf to reef transect from the Zaris Subbasin. We seek to quantify the ecophenotypic response of *Cloudina* tube construction to the environmental conditions of growth, by comparing whole tube sinuosity (the degree of curvature), as well as individual lamina thickness and overall tube wall thickness to test whether these are variable and hence subject to environmental control, or invariant and therefore under strong biological control. The sinuosity of *Cloudina* tubes, for example, is noticeably variable throughout the Nama Group, and this may reflect some aspect of ambient hydrodynamics or feeding efficiency. We also consider the control of differing early diagenetic style on the preservation of *Cloudina* skeletal material, as the inner ramp settings are prone to meteoric diagenesis but the more energetic mid-ramp reef settings to high carbonate supersaturation and marine phreatic diagenesis. Features found consistently across all diagenetic settings might be considered more likely to have a biological, rather than diagenetic, origin. We use descriptive and quantitative data derived from reef surfaces, and bedding planes, using petrographic and cathodoluminescence (CL) microscopy, and electron microprobe analysis (EMPA) of Sr concentrations, which can indicate differences in original carbonate mineralogy, and Mg/Ca which may also vary according to diagenetic phase or with biological fractionation.

## Geological setting

**Figure 1:**
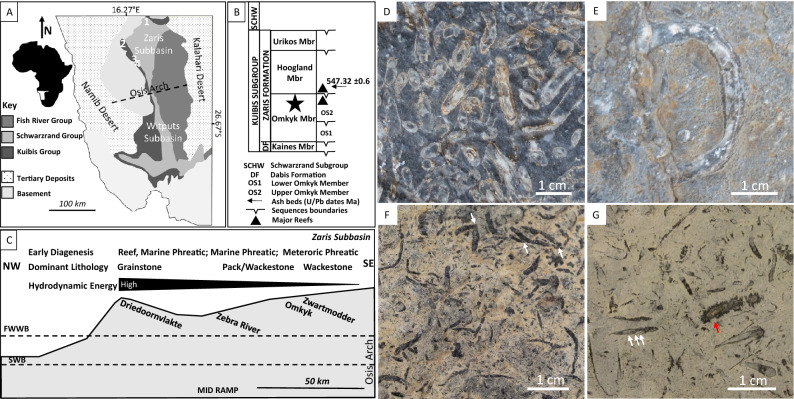
Geological map and study site locations (1: Driedoornvlakte; 2: Zebra River; 3: Omkyk Farm; 4: Zwartmodder) within the Nama Group, Namibia, with bedding surface images of *Cloudina* from different coeval communities from the Upper Omkyk Member (drawn in Microsoft PowerPoint 2016). (**A**) Localities, modified from Grotzinger and Miller 2008^21^. (**B**) Stratigraphy of the Nama Group with the Upper Omkyk Member highlighted by a star, modified from Wood et al.^25^ (drawn in Microsoft PowerPoint 2016). (**C**) Schematic of the Zaris Subbasin with relative position of localities, and dominant hydrodynamic regime and lithologies, and early diagenetic setting. Modified from Wood et al.^25^ (drawn in PowerPoint 2016). (**D**) Driedoornvlakte, preserved as white or grey calcite cement surrounded by darker calcite cements, predominantly pseudomorphed aragonitic botryoids. (**E**) Zebra River, infilled with light sparry calcite cement surrounded by dolomitised micrite. (**F**) Omkyk Farm, cloudinomorphs, probably *Cloudina*, including potential branching individuals (arrowed) as branching can only be proven through the presence of a shared cavity, preserved as black sparry calcite surrounded by dolomitised wackestone^7^. (**G**) Zwartmodder, preserved as black sparry calcite surrounded by dolomitised micrite which preserves the fine annulated structure and phlanges (white arrowed). Possible fragments of *Corumbella* may also be present (red arrow). Figure created in PowerPoint 2016.

The Nama Group, Namibia (ca. 550–539 Ma), is a late Ediacaran fossiliferous carbonate-siliciclastic succession deposited in supratidal to outer ramp settings^21^ (Fig. 1). The Zaris and Witputs Subbasins are separated by the tectonic Osis Arch, and are correlated using sequence stratigraphy and chemostratigraphy^22–24^. We consider *Cloudina* assemblages attributed to both *Cloudina riemkeae* and *Cloudina hartmannae* from four localities that provide a transect of the Upper Omkyk Member, Kuibis Subgroup across the Zaris Subbasin (Fig. 1B,C), from Driedoornvlakte (mid-ramp, high-energy reef), Zebra River (inner mid-ramp, thrombolitic-stromatolitic reefs), Omkyk (inner-ramp, low-energy), to Zwartmodder (proximal inner-ramp, very low-energy)^25^. These record coeval communities that grew at different water depths, with differing host lithologies, hydrodynamic energies, and early diagenetic settings (Fig. 1C; summarised in Table S1 and Supplementary Information). Ash bed U/Pb data collected from the overlying Hoogland Member has been dated at 547.32 ± 0.65 Ma^26^.

## Results

### Sinuosity of *Cloudina*

**Figure 2:**
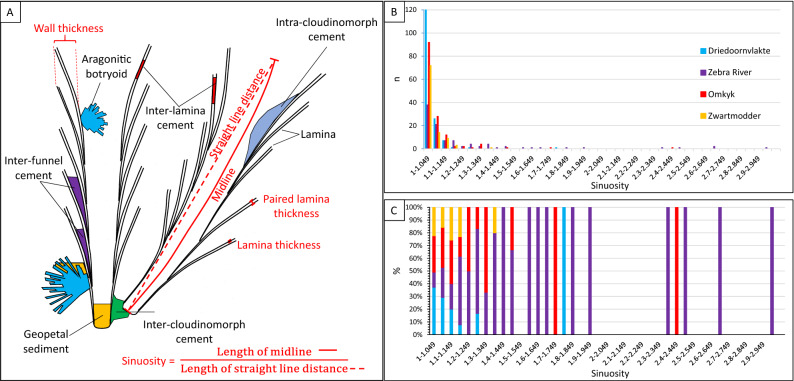
*Cloudina* terminology and sinuosity data. (**A**) Schematic of two *Cloudina* tubes with terminology (black text) and measurements (red text) used in this paper derived from 2D surfaces (drawn in Microsoft PowerPoint 2016). Sinuosity is defined as the length of the midline over the straight line distance between the two ends of the tube in longitudinal section. *Lamina thickness* is the thickness of an individual lamina and *paired laminae thickness* the thickness of two laminae including the area between. *Wall thickness* is the sum of all components that form the complete tube wall in transverse section from outer wall to inner wall defining the central cavity. Cements formed between paired laminae are termed *inter-lamina cement*, between sets of paired laminae *inter-funnel cement,* between different tubes *inter-cloudinomorph cement,* and that which forms within the central cavity is termed *intra-cloudinomorph cement*. Inorganic pseudomorphed aragonitic botryoidal cements and sediment have also been labelled in the figure. (**B**) Sinuosity distribution (created in Microsoft Excel 2016). (**C**) Percentage of *Cloudina* with different sinuosities, based on 2D bedding plane measurements. Figure created in PowerPoint 2016, graphs created in Microsoft Excel 2016.

The four localities show a varying degree of sinuosity of *Cloudina* tubes (Figs. 1D–G, 2B,C). At Driedoornvlakte, sinuosity ranges between 1.00 and 1.70 (mean = 1.04, n = 156, standard deviation = 0.07), with the percentage shortening (the amount of shortening as a percentage in relation to the length along the midline) ranging between 0.00 and 41.29% (mean = 3.23%, standard deviation = 4.42). Zebra River and Omkyk both show greater tube sinuosity, from 1.00 to 2.98 (mean = 1.21, n = 98, standard deviation = 0.38) and 1.00–2.42 (mean = 1.07, n = 144, standard deviation = 0.15), respectively. Zebra River *Cloudina* show shortening from 0.00 to 66.69% (mean = 13.01%, standard deviation = 15.8), greater than the percentage found at Omkyk, where the shortening ranges between 0.00 and 58.67% (mean = 5.22%, standard deviation = 7.86). Zwartmodder *Cloudina* shows the lowest range of sinuosity, from 1.00 to 1.36 (mean = 1.04, n = 99, standard deviation = 0.06), and shortening between 0.00 and 26.25% (mean = 3.27, standard deviation = 4.57). Comparing the percentage of different ranges of sinuosity values at each locality, *Cloudina* at Zebra River show the greatest sinuosity, accounting for 100% of these values (Fig. 2C). These also have the lowest percentage of low sinuosity values (12%), whereas *Cloudina* from Driedoornvlakte show the highest percentage of low sinuosity values (37%) (Fig. 2C).

Using the Kruskal–Wallis Test, the H-value for these data is 14.96, with a *P*-value of 0.002, indicating that the sinuosity of the *Cloudina* tubes varies significantly between localities. Z-tests also confirm that the sinuosity varies between all localities except between Driedoornvlakte and Zwartmodder.

### Diagenetic preservation of *Cloudina*

The form of preservation of *Cloudina* varies across the Zaris subbasin transect (Table S2).

**Figure 3:**
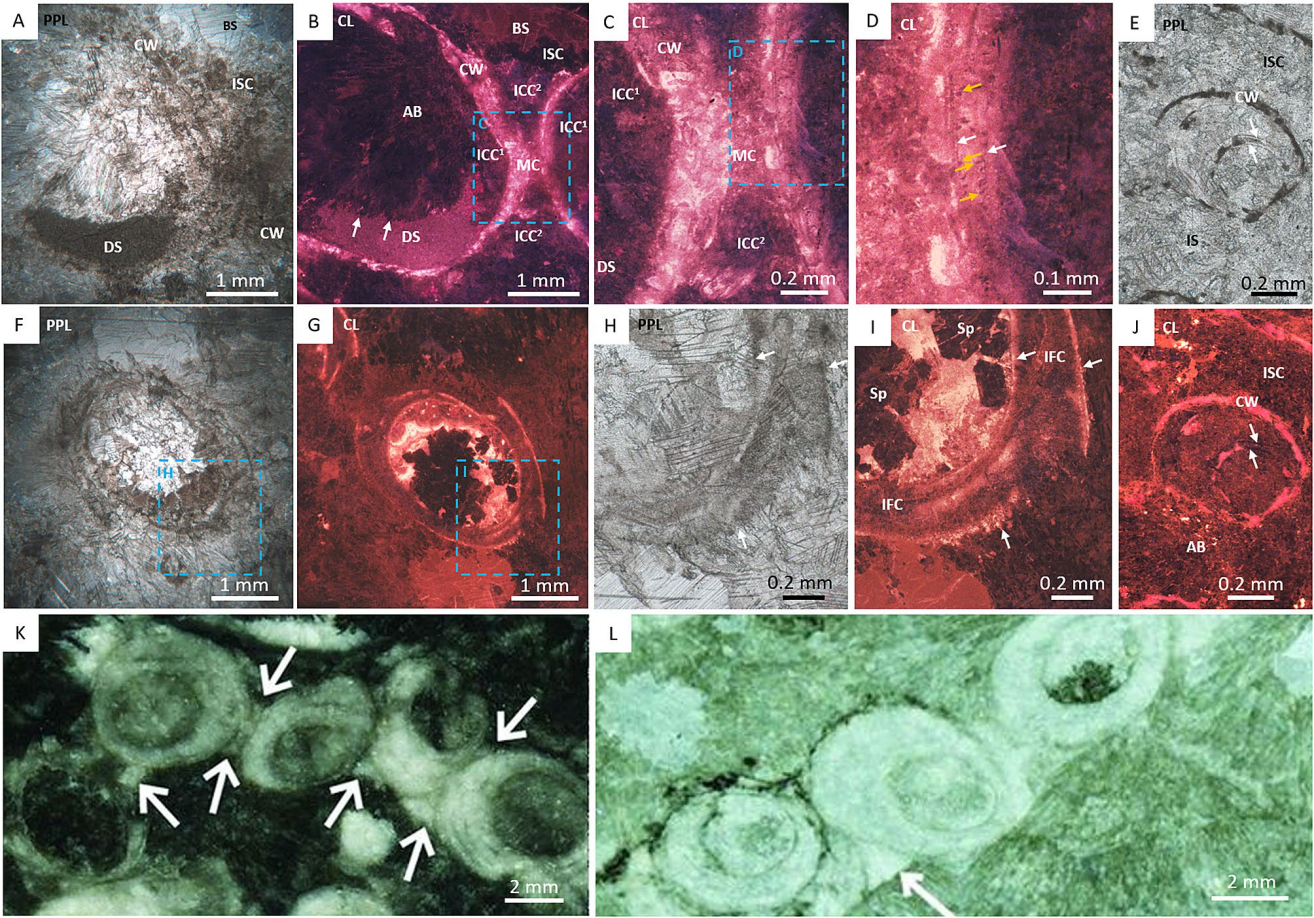
Photomicrographs of *Cloudina hartmannae* and *Cloudina riemkeae* from Driedoornvlakte, Upper Omkyk Member, Nama Group. (**A**) PPL of attached *Cloudina* with a dolomitised wall (CW) and filled with dolomitised sediment (DS), followed by an inclusion-rich sparry calcite (ISC) and a later clear burial spar (BS). (**B**) CL image of A, intra-cloudinomorph cement (ICC^1^) nucleated from the inner *Cloudina* wall and inter-cloudinomorph cement (ICC^2^) formed between the *Cloudina* tubes and nucleated from the outer wall and including the mutual cement (MC) which has the same luminescence as CW. Non-luminescent pseudomorphed aragonitic botryoids (AB) nucleate from ICC^1^. These are proceeded by ISC and BS. (**C**) Inset of B, showing ICC^1^ and ICC^2^ -cements with same luminescence. (**D**) Inset of C, showing bright luminescent CW with multiple non-luminescent laminae (white arrows) with small aragonitic needles (orange arrows) nucleating from laminae. (**E**) PPL of *Cloudina* with a dolomitised wall (CW) composed of laminae (arrowed) with thin sparry calcite infill between the two laminae, surrounded by inclusion-rich sparry calcite (ISC). (**F**) Transverse PPL of dolomitised individual. (**G**) CL of C. (**H**) Inset of F, of broken, dolomitised *Cloudina* wall (arrowed). (**I**) Inset of G in CL, of broken bright luminescent *Cloudina* wall (arrowed) exposing the inter-funnel cement (IFC). The rest of the tube is infilled with non-luminescent spar (Sp). (**J**) CL of E, with dull luminescent cements (arrowed) highlighting cements between the laminae and is surrounded by ISC. AB nucleate from the CW. (**K**) from Penny et al.^27^. Polished slab highlighting inter-cloudinomorph cements (arrowed) between multiple *Cloudina* individuals. (**L**) From Penny et al.^27^. PPL image of inter-cloudinomorph cements (arrowed) situated between *Cloudina* individuals from which botryoids nucleate. Figure created in PowerPoint, 2016.

At Driedoornvlakte, *Cloudina hartmannae* are often surrounded by pseudomorphed aragonitic botryoids^3,7,27^ (Fig. 1D). Individuals show brittle deformation. *Cloudina* walls are preserved as light grey calcite, or light brown to yellow crusts indicating partial dolomitisation (Fig. 1D). This inclusion-rich dolomite is brightly luminescent varying between bright yellow to red under CL (Fig. 3). Laminae are often found as pairs, where abundant non-luminescent fine acicular cements with blunt terminations (4.2–17.6 µm in length, mean = 11.2 µm; and 2.1–7 µm in width, mean = 4.5 µm, n = 17) that nucleate from the laminae (Fig. 3D). This is here termed *inter-lamina cement* (Fig. 2A). Between separate paired laminae is a patchily luminescent cement (Fig. 3I)—here termed *inter-funnel cement* (Fig. 2A). The paired laminae with inter-lamina cement, and inter-funnel cements constitute the *Cloudina* wall extending from the outer wall to inner wall that forms the central cavity (Fig. 2A).

A further dull, patchily luminescent cement is present, which formed from the innermost laminae and grew upon the inner wall of the tube—here termed *intra-cloudinomorph cement* (Figs. 2A, 3B,C;). A cement with similar cathodoluminescence is found between the tubes themselves—here termed *inter-cloudinomorph cement* (Figs. 2A, 3B,C,K,L), as noted by Penny, et al^27^. Dolomitised geopetal micrite with red luminescence infills *Cloudina*, and postdates the intra-cloudinomorph cement (Fig. 3A–C). Pseudomorphed aragonitic botryoids, with blunt crystal terminations, nucleate from both the intra- and inter-cloudinomorph cements, and are also covered by geopetal sediment (Fig. 3B). Breakage of laminae is evident, with associated breakage of the inter-funnel cement (Fig. 3D,F–I).

**Figure 4:**
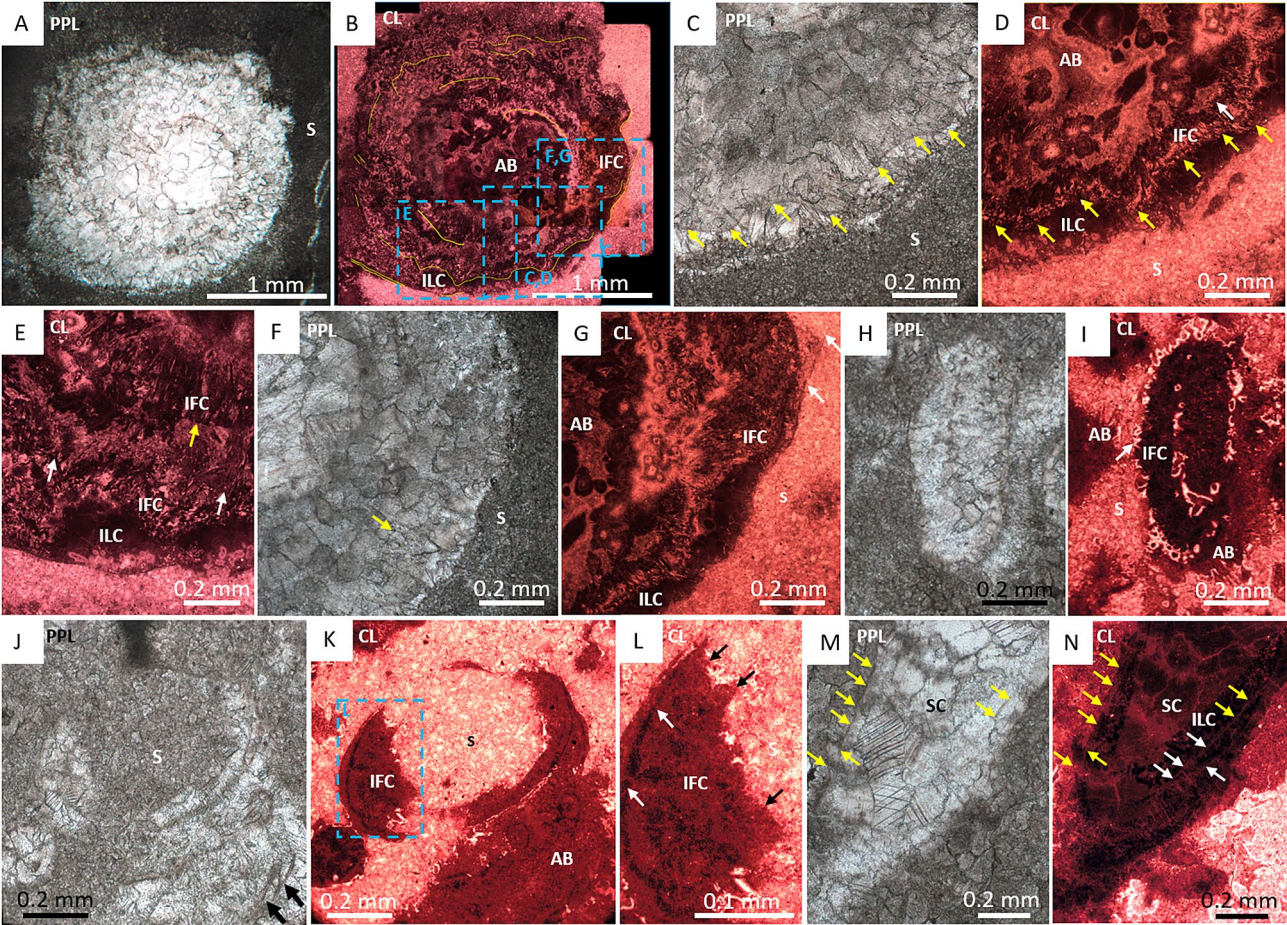
Photomicrographs of *Cloudina* from Zebra River, Upper Omkyk Member, Nama Group. (**A**) PPL of *Cloudina* infilled with sparry calcite surrounded by micritic sediment (S). (**B**) CL image of A, with inter-lamina cements (ILC) forming between outer laminae (highlighted in yellow). Pseudomorphed aragonitic botryoids (AB) grow from the laminae, and the inter-funnel cements (IFC) pre-date dull luminescent burial cements with ILC forming between paired laminae. AB infilled the *Cloudina* tube and S surrounds the tube. (**C**) Inset of B \, PPL showing laminae with a wavy form (yellow arrows). (**D**) CL image of E, laminae highlighted by yellow arrows with same form as PPL image. Nucleation of IFC indicates location of laminae. (**E**) Inset of B, ILC located between two outer laminae (white arrows). IFC nucleate from inner laminae (yellow arrow). (**F**) Inset of B in PPL with lamina visible (arrowed). (**G**) CL of F, where outer laminae shows evidence of ‘spalling’ (arrowed) and infilled by S. ILC situated between laminae and areas of patchy bright luminescence indicate IFC. AB infills the rest of the tube. (**H**) PPL of *Cloudina*. (**I**) Pseudomorphed aragonitic botryoids (AB) outside *Cloudina* tube. Botryoids grow from the outer tube wall into the surrounding sediment (S), and have a different luminescence to those inside the tube. Early IFC grows from the inner *Cloudina* laminae (arrowed). (**J**) PPL of a transverse section of a *Cloudina*, which is infilled by dolomitised sediment (S), and have a different luminescence to those inside the tube. Early IFC grows from the inner *Cloudina* laminae (arrowed). (**J**) PPL of a transverse section of a *Cloudina*, which is infilled by dolomitised sediment (S) and two sets of broken paired laminae (arrowed) (**K**) CL of J, bright luminescent dolomitised sediment infills the central area of the broken tube. Dull luminescent botryoids (AB) outside the tube. Dull luminescent IFC forms the *Cloudina* skeleton. (**L**) Inset of K, with evidence of breakage of the non-luminescent ILC (white arrows) and IFC (black arrows). (**M**) PPL of *Cloudina* tube infilled with sparry calcite (SC) with preserved laminae (yellow arrowed). (**N**) CL of M, with laminae observed in PPL (yellow arrows), laminae shown in CL (white arrows). Laminae are preserved with the same luminescence as the SC infill with a dull cement ILC between the laminae. Figure created in PowerPoint 2016.

*Cloudina* from Zebra River are preserved as calcite surrounded by wackestone (Fig. 1E). The ornamental features of the tube are not preserved and the tube cavity is infilled with sparry calcite. Individuals show brittle and ductile deformation. Paired laminae (Figs. 4K-L) are present, and the inter-lamina space is infilled by acicular crystals (Fig. 4D,G,N) ranging from 9.4 to 15.5 µm in length (mean = 11.7 µm, n = 6) and 2.1 to 4 µm in width (mean = 3.1 µm), forming the inter-lamina cement. In some areas the laminae are undulating, with a prominent inter-funnel cement infill (Fig. 4C–G,J–L).

Patchily luminescent inter-funnel cement is also present, formed by abundant clusters of acicular cements with blunt terminations which nucleate from the laminae (Fig. 4B,D,E,G). A dull luminescent, pore-lining patchy cement (Fig. 4C–G) overlies inter-funnel cements. Later tube cavity infill consists of calcite spar with duller luminescence (Fig. 4B). In *Cloudina riemkeae*, the acicular cements are finer and pore-lining cements are not observed, but inter-funnel cements with dull luminescence are present, followed by more luminescent pseudomorphosed aragonitic botryoids which nucleate from the outer tube walls (Fig. 4H–I). There is variation in the preservation of this inter-funnel cement, which varies even within a single *Cloudina* tube: inter-funnel cements close to the exterior of the *Cloudina* tubes are mostly preserved as clear, dull or non-luminescent neomorphic calcite spar, but inter-lamina cements within the central areas of the tubes preserve acicular crystals (Fig. 4C,D).

The inter-funnel cements in *Cloudina riemkeae* show breakage, with fractured crystal boundaries where sediment has entered the tube (Fig. 4J–L). Individual or paired laminae also show spalling as a result of breakage of the outer wall (Fig. 4G).

**Figure 5:**
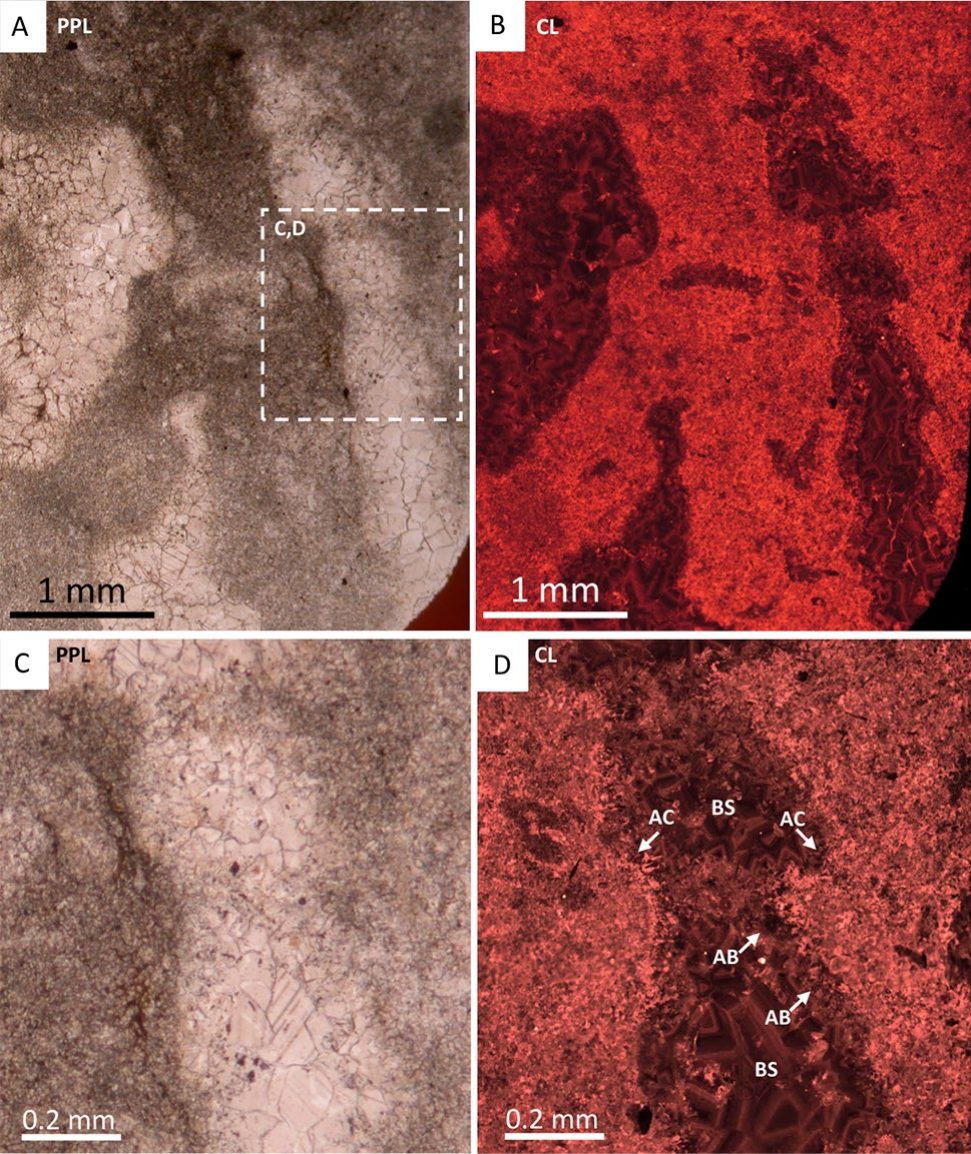
Photomicrographs of cloudinomorphs, probably *Cloudina,* from Omkyk Farm, Upper Omkyk Member, Nama Group. (**A**) PPL image of cloudinomorph tube. (**B**) CL image of A. (**C**) Inset of A. (**D**) CL image of A, with early acicular cements (AC) which vary between bright and non-luminescence. AC is followed by poorly-zoned acicular bladed calcite (AB). A well zoned blocky calcite (BS) infills the remaining tube cavity. Figure created in PowerPoint 2016.

The cloudinomorphs of Omkyk are dark in colour and composed of large (0.1–1 mm) calcite spar crystals (Fig. 1F). These also lack ornamentation, but some retain the stacked funnel-in-funnel structure^7^. Individuals show brittle and ductile deformation. CL highlights three generations of cements within the cloudinomorph tube (Fig. 5A,B,D). First a thin (200 µm) acicular cement generation, which is not continuous throughout the tube cavity, followed by an isopachous cement with patchy or dull-luminescence with limited zonation (Fig. 5D). The tube cavity is infilled by zoned sparry cement, which nucleates from the isopachous cement (Fig. 5D).

**Figure 6:**
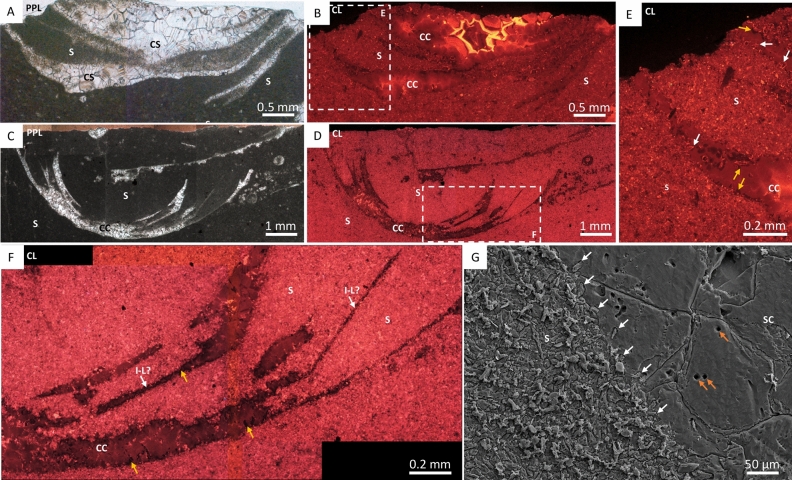
Photomicrographs of *Cloudina* from Zwartmodder, Upper Omkyk Member, Nama Group. (**A**) PPL of compacted *Cloudina* skeleton (CS) surrounded by dolomitised micritic sediment (S). (**B**) CL of A, featuring dull cement which becomes well-zoned with bright luminescence during later growth. The cement infill is centripetal cement (CC) which nucleates from the wall and grows into the mould formed through dissolution. (**C**) PPL of broken and spalled laminae. (**D**) CL of C, the skeletal tube consists of a dull luminescent CC, which in thicker areas is brightly luminescent. Dolomitised micritic sediment surrounds the `spalling' skeleton. (**E**) Inset of B, brightly luminescent cement nucleates from sediment grains, which protrude into the *Cloudina* mould (white arrows) and are overgrown by a non-luminescent cement (yellow arrows) that formed before CC. (**F**) Inset of D, sediment infills areas between spalling potential inter-laminae (I-L?), spalling occurs where CC has not formed between the laminae. Bright luminescent cements on micritic sediment grains which protrude into the *Cloudina* mould which is overgrown by a non-luminescent cement (yellow arrows). (**G**) Sparry calcite (SC) infill of the *Cloudina* skeleton, where cement crystals protrude from surrounding sediment (white arrows). Cube-shaped holes in the SC may represent plucked micro-dolomite or pyrite crystals. Figure created in PowerPoint 2016.

Zwartmodder *Cloudina* are also preserved wholly as coarse calcite spar (Figs. 1G, 6A,C). Fine external features, including external phlanges and the annulated outer wall, are well preserved (Fig. 1G), and *Cloudina* shows evidence of brittle deformation (Fig. 6C). The *Cloudina* skeleton is preserved a mould, infilled by a centripetal, sparry calcite cement with dull luminescence that becomes brighter towards the centre of the moulds (Fig. 6A,B). Individual laminae cannot be detected, but inter-lamina and inter-funnel cement are present as sparry calcite (Fig. 6F). Individual or paired laminae are evident through the spalling of the tube walls in areas where inter-funnel cement is not present and are instead separated by micrite infill (Fig. 6F). Under CL, the micrite shows three zones of cement growth, dull-luminescent followed by bright luminescent, and a final non-luminescent zone (Fig. 6B,E,F). These cement zones protrude into the sparry calcite infill, which can also be seen under SEM along with small, cube shaped holes (Fig. 6G).

### Elemental distribution

**Figure 7:**
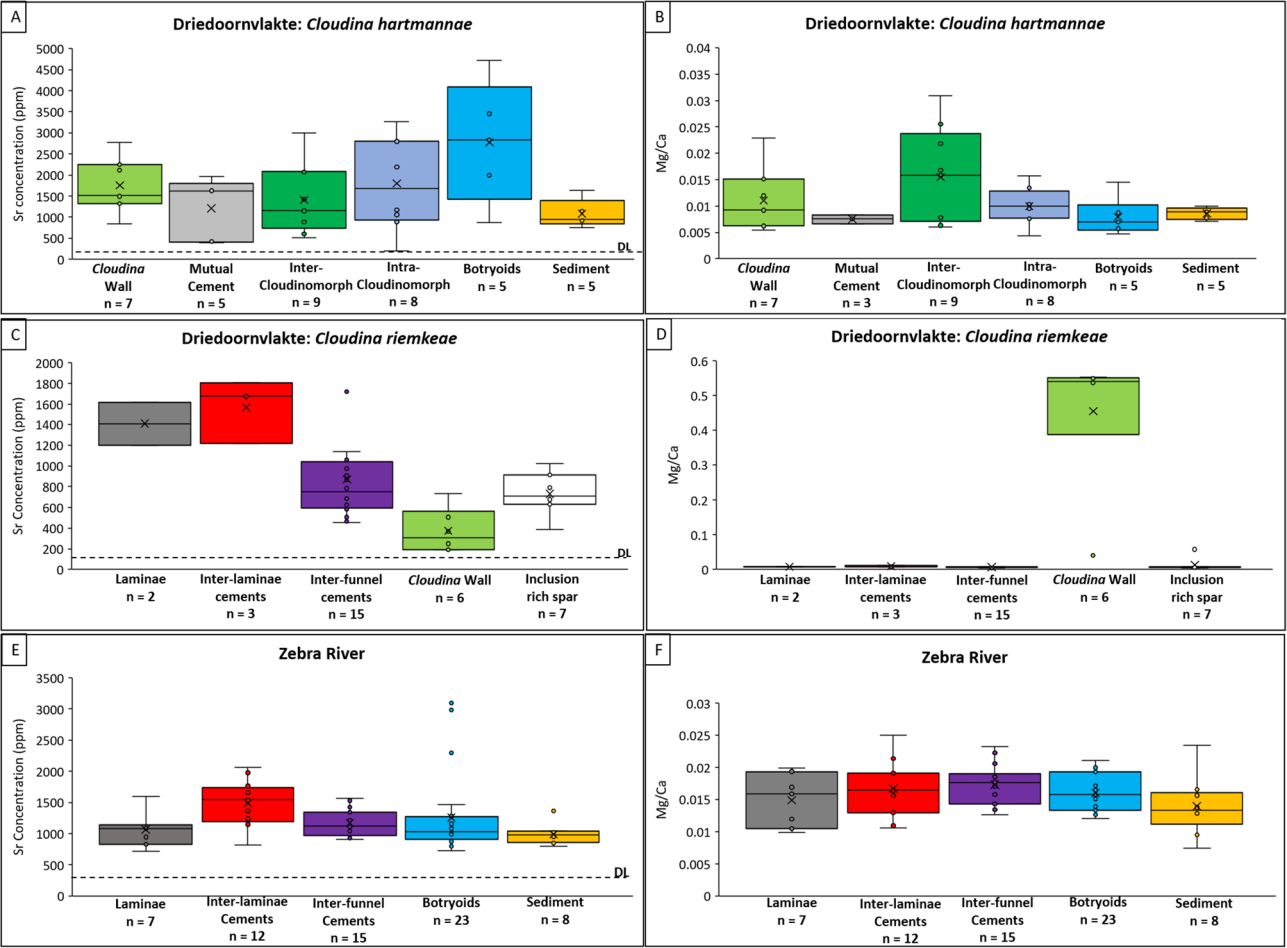
EMPA data from *Cloudina* and associated cements from Driedoornvlakte and Zebra River, Upper Omkyk Member, Nama Group. (**A**) Strontium concentration (ppm). (**B**) Mg/Ca ratio of *Cloudina riemkeae* from Zebra River. (**C**) Strontium concentration (ppm) of *Cloudina hartmannae* from Driedoornvlakte. (**D**) Mg/Ca ratio of *Cloudina hartmannae* from Driedoornvlakte. E: Strontium concentration (ppm) of *Cloudina riemkeae* from Zebra River. (**F**) Mg/Ca ratio of *Cloudina riemkeae* from Zebra River. Detection limits for elements are shown as horizontal dashed lines (DL). Figure created in PowerPoint 2016, graphs created in Excel 2016.

Strontium (Sr) concentrations and the Mg/Ca ratios were sampled from *Cloudina* and associated early cements from Driedoornvlakte and Zebra River using EMPA (See Methods, Supplementary Information; Fig. 7; S1; Tables S3–S6).

The Kruskal–Wallis test shows a statistical difference between the Sr concentration of different cements at Zebra River, between the inter-laminae cements and both laminae (*P* = 0.016) and inter-funnel cements (*P* = 0.014), and between the micritic matrix and both inter-laminae cement (*P* = 0.001) and inter-funnel cement (*P* = 0.030) (Table S7). There are no statistical differences in Mg/Ca contents of any measured features (Table S8).

*Cloudina riemkeae* from Driedoornvlakte also shows statistical differences in Sr concentration between the inter-laminae and the inter-funnel cements (*P* = 0.041) (Table S9), and between inter-laminae and the inter-funnel cements compared to the *Cloudina* wall (*P* = 0.009 and *P* = 0.002, respectfully) (Table S9). Sr content of the inter-laminae cement and *Cloudina* wall are statistically different to the inclusion-rich spar (*P* = 0.023 and *P* = 0.011, respectfully), but this is not the case for the inter-funnel cement (*P* = 0.291) which is recrystallised as an inclusion-rich spar (Fig. 3E,J; Table S9). There is a significant difference between the *Cloudina* wall at Driedoornvlakte and the other *Cloudina*-associated cements, probably as a result of the dolomitisation. Sr content within internal cements associated with *Cloudina hartmanna*e show no statistical differences, but there is a significant difference between the inter-cloudinomorph cement and botryoidal cements (*P* = 0.043) (Table S10).

There are no statistical differences in Mg/Ca contents of any measured features at Driedoornvlakte apart from the *Cloudina* wall of the *Cloudina riemkeae*, due to selective dolomitisation (Tables S11, S12).

### Lamina thickness

Organic laminae have not been preserved at the studied sites. Laminae are preserved as moulds at Zebra River and Zwartmodder (Figs. 4, 6), at Driedoornvlakte they have been dolomitised (Fig. 3), whilst at Omkyk laminae are not detectable at all (Fig. 5). Laminae thickness at Zebra River and Driedoornvlakte were measured using both CL and PPL images and using CL at Zwartmodder.

**Figure 8:**
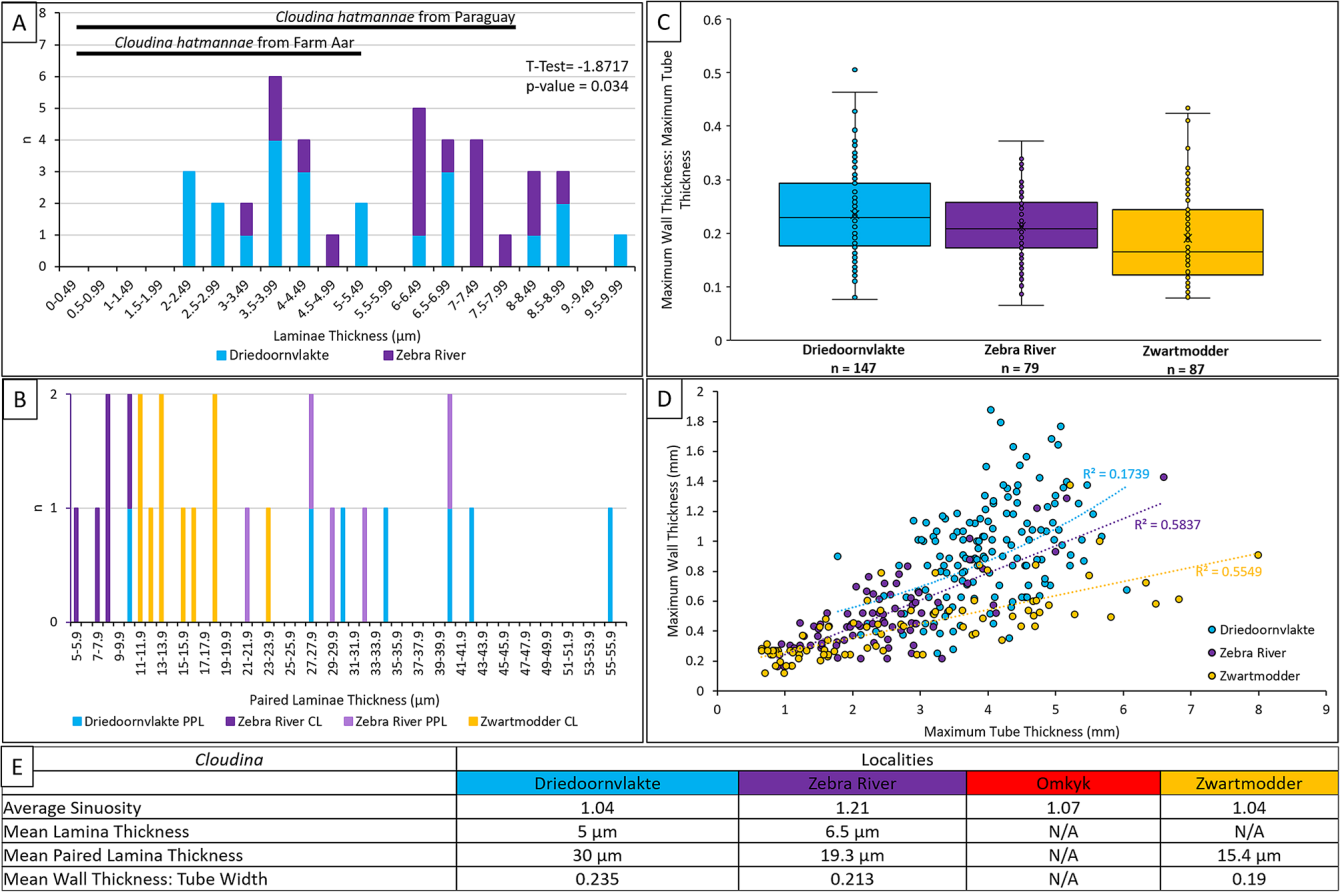
Features of *Cloudina* walls, including laminae thickness, paired laminae thickness and ratio between the maximum *Cloudina* wall width and maximum tube width of *Cloudina* of the Upper Omkyk Member, Zaris Subbasin, Namibia. (**A**) Lamina thickness, with thicknesses reported from Yang et al.^15^. (**B**) Paired laminae thickness. (**C**) Distribution of ratios of wall thickness: tube thickness. (**D**) Relationship between wall thickness: tube thickness. (**E**) Summary of quantitative features of *Cloudina* at the four coeval sites of the Upper Omkyk Member. Figure created in PowerPoint 2016, graphs created in Microsoft Excel 2016.

*Cloudina* of Driedoornvlakte and Zebra River have laminae of similar thicknesses, with Driedoornvlakte laminae thicknesses ranging from 2.1 to 9.6 µm (mean = 5 µm, n = 23) and Zebra River *Cloudina* laminae ranging from 3.3 to 10.8 µm in thickness (mean = 6.5 µm, n = 23), with a T = 1.872, and *P*-value = 0.034 (Fig. 8A).

Paired laminae thickness, however, varies between sites. At Driedoornvlakte from 10.6 to 55.3 µm (mean = 34.3 µm, n = 14), Zebra River from 5.9 to 40.3 µm (mean = 19.3 µm, n = 10), and Zwartmodder from 11 to 23.7 µm (mean = 15.4 µm, n = 10) (Fig. 8B). The spalled features of the *Cloudina* from Zwartmodder are now preserved as centripetal calcite cement (Fig. 6F) and consist of the inter-lamina cement and two laminae; the thickness of these features is similar to that of the paired laminae at Driedoornvlakte and Zebra River.

These differences are statistically significant (T = 2.32, *P*-value = 0.040). The Kruskal–Wallis Test shows that paired laminae thickness from Driedoornvlakte, Zebra River and Zwartmodder, are significantly different (H-value = 7.65, *P*-value = 0.022).

### Wall thickness

*Cloudina* wall thickness is defined as the width between the outer tube wall and the inner tube wall forming the internal cavity (Fig. 2A). Measurements of wall thickness were taken from bedding surfaces at Driedoornvlakte, Zebra River and Zwartmodder. At Driedoornvlakte, maximum wall thickness for any individual *Cloudina* tube ranges from 0.25 to 1.88 mm (mean = 0.93 mm, n = 147) (Fig. 8C). Ranges were lower at Zebra River and Zwartmodder, from 0.16 to 1.43 mm (mean = 0.51 mm, n = 79) and 0.12–1.38 mm (mean = 0.42 mm, n = 87), respectively (Fig. 8C).

When comparing maximum wall width: maximum tube width, *Cloudina* at Driedoornvlakte show the greatest ratios ranging from 0.077 to 0.505 (mean = 0.235), with Zwartmodder ranging from 0.079 to 0.434 (mean = 0.190), and Zebra River showing the smallest range from 0.065 to 0.375 (mean = 0.235) (Fig. 8C). All localities show a weak positive correlation between the thickness of the tube and the maximum wall thickness (Fig. 8D). Ratios are statistically different between Driedoornvlakte and both Zebra River and Zwartmodder (*P* = 0.031 and *P* = 5.77^–5^, respectively), but not between Zebra River and Zwartmodder (*P* = 0.060).

## Discussion

### Sinuosity of *Cloudina*

*Cloudina* at Driedoornvlakte and Zwartmodder show the lowest sinuosity, and Zebra River the highest (Fig. 2B,C). This systematic variation (Table S2; Fig. 8E) suggests that this feature is in some way environmentally dictated, and that mineralisation of *Cloudina* occurred via a flexible organic templates that allowed adaptation to local conditions. Very little is known as to the controls on sinuosity in modern benthos, but orientation to maximise feeding efficiency in ambient currents and local sedimentation regime is thought to be a major control in calcareous tubed polychaetes (serpulids)^28,29^. Creating hypotheses as to what advantage might be conferred by increased sinuosity is therefore problematic, but sinuosity may be governed by diverse factors such as substrate type and morphology, vertical or horizontal growth, competition for space, nutrient regime, water depth, and response to hydrodynamic energy and water flow.

We note that sinuosity measurements are based on 2D bedding plane measurements, and further insight might be gained by 3D analysis for both more accurate sinuosity quantification, as well as information about curvature in the third dimension.

### Diagenesis of *Cloudina* and associated cements

While local diagenesis controls the expression of laminae, ranging from dolomite replacement of another carbonate phase, to moldic preservation, i.e. dissolution of an unstable carbonate phase, individual lamina thickness is always consistent (Table S2). We have not observed the micritic microstructure described for the walls of *Cloudina*, but these modes of preservation are consistent with laminae being organic-rich but calcified.

Moldic preservation at Omkyk and Zwartmodder has resulted in the absence of preserved skeletal walls. Early acicular isopachous cement generations are present at Omkyk, which grew from the cloudinomorph walls, followed by an infilling well-zoned clear sparry burial calcite^7^ (Fig. 5). *Cloudina* are preserved only via a sparry infill of moulds at Zwartmodder, which represent dissolved paired laminae together with inter-lamina cement and inter-funnel cement (Fig. 6). Dissolution pre-dated cement growth around the grains of the surrounding sediment as this cement grew into *Cloudina* moulds (Fig. 6E).

The extent of dissolution at these shallow, inner ramp localities suggest the influence of freshwater via early meteoric diagenesis, perhaps associated with the degradation of the organic material, which would preferentially remove aragonite. By contrast, the mid-ramp sites Driedoornvlakte and Zebra River, preserve very early botryoidal pseudomorphed aragonitic cements formed in cavities and pores within the marine phreatic zone.

Zebra River *Cloudina* show undulose laminae, with some evidence of both brittle fracturing and ductile deformation (Fig. 4G), in contrast to Driedoornvlakte where there is only evidence of brittle deformation (Table S2). Compaction also caused breakage of *Cloudina* tubes at Zebra River and Zwartmodder, similar to that of a spalling ooid, where the resultant area was infilled by sediment (Figs. 4G, 6F). Inter-lamina and inter-funnel cements formed prior to the breakage of the *Cloudina* tubes, and that of the laminae, where moulds were later filled by a burial cement (Fig. 4M,N). The formation of the dull luminescent inter-funnel cement, seen most clearly at Zebra River, occurred before compaction and lithification of the surrounding dolomitised sediment as evidenced by the sharp fracture of the inter-funnel cement where sediment has encroached into the tube (Fig. 4J–L). All these cements also formed prior to the precipitation of pseudomorphed aragonitic botryoidal cements both inside and outside the tubes (Figs. 3B, 4B). Botryoids external to *Cloudina* tube have brighter luminescence than those within the tube, suggesting some degree of diagenetic compartmentalisation. At Zebra River, the pseudomorphed aragonitic acicular crystals are not preserved in the outermost inter-lamina cements, but rather were replaced by the non-luminescent neomorphic cements later in diagenesis.

Inter-lamina, inter-funnel, intra-cloudinomorph, and inter-cloudinomorph cements are all composed of fine, acicular crystal bundles (mean width = ca. 3–5 µm, length = ca. 11 µm) that nucleated on both *Cloudina* laminae and from the outer wall of the tube. Crystal terminations are blunt and are inferred to be pseudomorphed aragonite. All precipitated prior to transport and breakage of the tubes, and also pre-dated the cement botryoids, so can be inferred to be very early syn-sedimentary. The sparry calcite noted previously^15^ is either neomorphic or burial spar that formed after the replacement or dissolution of these original cements.

Inter-funnel cements, first described by Grant^3^ have also been documented in *Cloudina* from Brazil^14,30^, Paraguay^9^, and Spain^31^, suggesting that such cements are a widespread feature of *Cloudina* present irrespective of early diagenetic setting, mineralogy, or palaeogeographic region. These cements probably formed when *Cloudina* was in-situ and provided mechanical strength and rigidity to the tube.

These cements are similar to those described from the skeleton in the extant sphinctozoan sponge *Vaceletia*, suggested to have a basal mode of biomineralisation^32^. Here, the skeleton is secreted upon a non-collagenous organic template, which becomes substituted by crystalline aragonite deposited as tangled crystal bundles of aragonite. The organic framework consists of proteins and polysaccharides rich in galactose, glucose and fucose, the latter suggesting that bacterial EPS (exopolymeric substances) may be involved in calcification^33^. In most cases, the basal parts of the skeleton, which is free from living tissue, is infilled by a micritic granular secondary deposit. The presence of organic matter has led to the suggestion of biofilm or microbial involvement in such cement precipitation for *Cloudina*^14^. Similar secondary deposition can occur where aragonite crystals continue to grow after soft tissue has vacated a region of the skeleton. This is known in taxa as diverse as scleractinian corals^18^ and the algae *Halimeda*^19^. So it is not clear if these cements in *Cloudina* formed during life, or in-situ but post-mortem, or in parts of the *Cloudina* skeleton abandoned by soft-tissue.

### Elemental signatures

Elemental signatures of similar cements cannot be compared directly between localities because of differing diagenetic histories, but statistically significant differences between phases can be determined for each locality. First, we note statistical differences in Sr concentration between various *Cloudina-*associated cements and botryoidal cements. This potentially indicates that the *Cloudina-*associated cements were of a different origin. However, the botryoidal cement used for this comparison is located within *Cloudina* tubes, adjacent to intra-cloudinomorph cements and here there is no significant difference in Sr values (*P* = 0.197) (Table S10). When comparing the Sr content of inter-cloudinomorph cement to those measured from botryoids outside *Cloudina* tube^34^, no significant difference is found between these cements. This suggests that all cements found within the *Cloudina* tube irrespective of type retain a higher concentration of Sr compared to those cements situated outside tubes, where leaching was more extensive. This is supported by the higher mean Sr concentration of the intra-cloudinomorph cement compared to the inter-cloudinomorph cement. We find no statistical differences between the *Cloudina*-associated cements, the inorganic botryoids and dolomitised sediment, which suggests that they cannot be distinguished using this criterion.

A similar conclusion was reached from study of Sr content of *Cloudina* from the Tamengo Formation of the Corumbá Group, Brazil^14^. On the basis of their timing of precipitation and the acicular, but non-botryoidal, texture, we conclude that all internal cements associated with *Cloudina* precipitated very early, but lack any distinctive Sr or Mg/Ca signature that might indicate either a diagenetic origin from a different pore fluid or biological fractionation.

### Lamina thickness

The variation of paired lamina thicknesses noted could be due to deformation between the laminae, especially at Zebra River, as laminae are observed to be flexible at this site. However, these differences are more likely due to the different methods used to measure paired laminae thickness: laminae at Zebra River and Driedoornvlakte were measured using both CL and PPL images, but the CL images show thinner laminae compared to their PPL counterparts (Fig. 8B). When comparing data of laminae thickness collected from PPL images only, the data sets are not statistically different (T = 0.57) and so the null hypothesis that the paired laminae thickness at Zebra River and Driedoornvlakte is the same is supported, but due to the small sample size this is not significant (*P*-value = 0.58). This is not the case, however, when comparing the CL data, as T-Test values indicate the paired laminae thickness varies (T = 2.54, *P*-value = 0.029), especially when spalled laminae are not included with the calculations (T = 4.75, *P*-value = 0.002). When comparing the thickness of the moldic-paired laminae seen at Zwartmodder, the values fall in the range of paired laminae at other sites. This suggests that the assumed moldic laminae are paired laminae combined with inter-lamina cements, as observed at Driedoornvlakte and Zebra River.

Presumed *Cloudina* laminae at Zwartmodder also occupy a narrower range of paired laminae thicknesses than those from Driedoornvlakte and Zebra River (mean = 15.4 µm). These laminae are expressed as sparry-calcite infilled moulds formed by the dissolution of both the paired laminae and the inter-lamina cement, and so this dissolution may account for the increased range of laminae thickness at Zwartmodder.

*Cloudina* laminae from the Mooifontein Member have a thickness of 0.5–5 µm, and samples from Paraguay range between 0.5 and 8 µm^15^, so falling within the overall range found in this study (Fig. 8A). Although we note greater lamina thicknesses, we consider these to be likely artefactual due to thickening by dolomitisation at Driedoornvlakte.

### Variability of wall thickness

The maximum thickness of the *Cloudina* wall and the thickness of the wall as a ratio of tube diameter is variable across the Zaris Subbasin (Fig. 8D,E). The weak positive correlation between the thickness of the tube and the maximum wall thickness at all localities suggests that the wall thickness was not a function of tube width. These data show that for a given tube width, the thickness of the wall is greatest at Driedoornvlakte, which is significantly and statistically greater than those at other sites.

This implies that wall thickness is environmentally-controlled, determining the distance between paired laminae sets and also potentially the volumetric extent of inter-lamina and inter-funnel cement formation. Driedoornvlakte was the most hydrodynamically energetic of those localities analysed, where rates of carbonate precipitation may have been higher, as shown by the abundant, volumetrically-significant syn-sedimentary botryoidal cements^27^. Such a regime may have promoted more rapid precipitation, and increased volumes, of internal cements. This is consistent with the observation that only brittle fracture is noted at Driedoornvlakte. Many other environmental parameters might have been important to produce a robust, more heavily calcified, and strong skeletal wall in this setting, however, such as enhanced food availability or as a response to currents.

## Conclusions

The consistent lamina thickness of *Cloudina* along the Zaris Subbasin shelf suggest that lamina formation was under biological control (Fig. 8E). The moldic or replacive dolomitised preservation of lamina indicate calcification of an organic-rich structure, potentially during life, from which early, acicular pseudomorphed aragonitic cements could nucleate. The precipitation of these cements pre-dates breakage prior to sediment infill, transport, and pseudomorphed aragonitic botryoid precipitation. The presence of such internal cements is a widespread feature of *Cloudina,* although diagenetic expression varies. Geochemical analysis (Mg/Ca; Sr concentrations), however, shows no statistically significant differences between these cements and the surrounding sedimentary matrix, and so no signature of biological fractionation is detected. We conclude that these cements associated with *Cloudina* formed rapidly, but it is not clear if they formed during life, post-mortem, or in parts of the *Cloudina* skeleton that were abandoned by soft-tissue as the animal grew to occupy younger parts of the skeleton. But the formation of these cements, particularly the inter-lamina and inter-funnel cements, would impart rigidity to the *Cloudina* tube, and the inter-cloudinomorph cements would create attachment between adjacent tubes.

The variation of sinuosity in *Cloudina* in different populations across the ramp of the Zaris Subbasin (Fig. 8E) implies that the curvature of the tube is environmentally-controlled, perhaps to maximise feeding efficiency in any given setting. This complements the findings that *Cloudina* tube diameter is also environmentally variable within the Nama Basin^10^. Variability in the cloudinomorph wall thickness is not a function of tube width and also differs between localities (Fig. 8E), further suggesting the influence of environmental factors in determining the distance between paired laminae sets and the volumetric extent of inter-lamina and inter-funnel cement formation. This may have been controlled by factors such as carbonate supersaturation or hydrodynamic energy, as thicker walls and only brittle fracture are noted in high-energy reef settings.

## Material and methods

ImageJ (Fiji) software (https://imagej.net) was used to quantify the size of features from photographs, hand specimens, and thin sections. Sinuosity, the degree of curvature, of the cloudinomorph tubes and wall thickness from bedding surface images was determined using ImageJ. Sinuosity, a term mostly associated with river morphology, is defined by dividing the length of an object by the length of the straight-line distance from bedding plane surfaces (see Fig. 2A). Values of < 1.1 indicate straight linear objects with higher values indicating increasing sinuosity. Percentage shortening is the amount of shortening of the tube in comparison to the original and assumed straight *Cloudina* tube and is calculated as a percentage ((straight line distance/ midline distance)*100/midline distance). A large number of measurements were obtained to overcome any systematic bias due to use of 2D measurements.

Highly-polished thin sections were used for plane polarised light (PPL) and cathodoluminescent (CL) petrography on a cathodoluminescence Cold Cathode CITL 8200 MK3A attached to a Nikon optiphot microscope at the University of Edinburgh. Samples from Zwartmodder were imaged using a Carl Zeiss SIGMA HD VP Field Emission scanning electron microscope (SEM) at the University of Edinburgh. Sections from Driedoornvlakte and Zebra River were used to quantify major element concentrations (Ca, Mg, Sr) of *Cloudina* and associated diagenetic components via Electron Microprobe analysis (EMPA) following CL images to test for differences in original mineralogy, diagenetic phase, or evidence of vital fractionation. EMPA was undertaken on a Cameca SX100 Electron Microprobe at the University of Edinburgh using a 80 s count time, a beam diameter of 3 µm, an accelerating voltage of 15 kV, and a beam current of 35 nA.

All data were statistically analysed using the Kruskal–Wallis Test, after data normalisation, using MS Excel 2016. Z-Tests were used on sample sizes where n > 50, such as sinuosity, and T-Tests were undertaken where sample sizes were n < 50 to provide a statistical comparison of each site using MS Excel 2016, variance was tested to determine which T-Test function to use, i.e. whether data sets had equal or unequal variance.

## Supplementary Information

Supplementary Information.
