# Supplementary material for: Environmental and diagenetic controls on the morphology and calcification of the Ediacaran metazoan Cloudina
Source: Sci Rep. 2021 Jun 11;11:12341. doi: 10.1038/s41598-021-90768-5 (PMC8195988; doi:10.1038/s41598-021-90768-5)
Supplement: Supplementary file 1 — Supplementary Information. [file 41598_2021_90768_MOESM1_ESM.docx]

**Supplementary Information: Environmental and diagenetic controls on the morphology and calcification of the Ediacaran metazoan *Cloudina***

Amy Shore and Rachel Wood

**Localities**

Driedoornvlakte is an energetic mid-ramp, shallow reef complex containing skeletal taxa, such as *Cloudina*, *Namacalathus* and *Namapoikia*. The Lower Omkyk Member is dominated by carbonate grainstones and the upper Omkyk Member by microbial reefs and stromatolites and thrombolites^1^. The first evidence of metazoan-supported reefs created by aggregating *Cloudina spp.* has been found within the Upper Omkyk Member of Driedoornvlakte^2^. *Cloudina* at Driedoornvlakte is often associated with as large (up to 100 mm in radius) syn-sedimentary aragonitic botryoidal cements^2^. The sampled *Cloudina* are from reef-derived material from bedding planes and tangential sections through beds

Zebra River represents a more proximal, mid-shelf ramp composed of grainstones in the Lower Omkyk Member and microbial mat, thrombolites and stromatolites, with grainstones and shales in the Upper Omkyk Member^3,4^. *Cloudina spp.* are attached to columnar and elongate thrombolites and are also found as inter-mound debris. *Namacalathus* is also found within the inter-column infill^5.^

The Omkyk Member of Omkyk Farm was deposited in a low energy, upper mid-ramp to inner ramp environment^4^. Dolomitised, finely laminated wackestone is abundant in the uppermost Upper Omkyk Member with evidence of potential microbial mats with associated, attached skeletal metazoans including polytomous-branching cloudinomorphs probably attributable to *Cloudina^6^*.

At Zwartmodder, the Omkyk Member consists of mainly dolostones at its base and grades to dominantly limestone laminites and packstones with grainstones at the top of the sequence^4^. The Upper Omkyk Member is composed of laminites and thrombolites and was deposited in a low energy, potentially, lagoonal setting within a highstand system tract, with abundant associated *Cloudina spp.* and *Namacalathus* ^4,6^.

## Elemental distribution

Strontium concentrations (ppm) and the Mg/Ca ratios were sampled from *Cloudina* and associated early cements from Driedoornvlakte and Zebra River using EMPA (Figs. 7; S1; Tables S3-12). Detection limit for Sr ranges from 148 – 117 ppm, mean = 133 ppm. Data are given to 4 decimal places.

At Driedoornvlakte, the Sr values of *Cloudina* laminae range from 777 to 1617 ppm (mean = 1199, n = 3) and Mg/Ca ratios from 0.007 to 0.367 (mean = 0.128, n = 3). Inter-laminae cements have a higher Sr content, from 1221 to 1804 ppm (mean = 1566, n = 3), and Mg/Ca values from 0.008 to 0.012 (mean = 0.009, n = 3). The Sr concentration of the inter-funnel cements vary greatly, even within the one sample, ranging from 457 to 1747 ppm (mean = 860 ppm, n = 16), with Mg/Ca ranging from 0.004 to 0.012 (mean = 0.007, n = 16). The degree of dolomitisation in samples makes differentiation between lamina, inter-laminae cements and inter-funnel cements difficult, so the term ‘*Cloudina* wall’ is used to describe the outer areas of the *Cloudina* tube where these cements are indistinguishable. The Sr values for *Cloudina* walls are highly variable, ranging between 189 and 2773 ppm (mean = 1119 ppm, n = 13), with Mg/Ca values ranging between 0.005 and 0.552 (mean = 0.216, n = 13). Values also vary between between *Cloudina riemkeae* and *Cloudina hartmannae*, with Sr data ranging from 189 to 736 ppm (mean = 373.83, n = 6) and 840 to 2773 ppm (mean = 1759 ppm, n = 7), respectfully. Intra-cloudinomorph and inter-cloudinomorph cements range in Sr concentration from 195 to 3267 ppm (mean = 1179 ppm, n = 24) and 521 to 3003 (mean = 1409 ppm, n = 9), respectively. The Mg/Ca of intra-cloudinomorph cements ranges between 0.008 to 0.016 (mean = 0.010, n = 8), with inter-cloudinomorph cements varying from 0.006 and 0.031 (mean = 0.015, n = 9).

At Zebra River, the Sr concentration of laminae varies between 719 and 1596 ppm (mean = 1064, n = 7), with Mg/Ca values ranging between 0.010 and 0.025 (mean = 0.017, n = 7). The Sr concentration in the inter-laminae cements have a wide range, from 819 to 1567 ppm (mean = 1490 ppm, n = 15), but the Mg/Ca values are more consistent, from 0.011 to 0.025 (mean = 0.017, n = 12). The inter-funnel cements have a more limited range of Sr values, from 904 to 1567 ppm (mean = 1169 ppm, n = 15), with Mg/Ca values ranging from 0.013 to 0.023 (mean = 0.017, n = 15).

The Sr values of pseudomorphed aragonitic botryoids at Driedoornvlakte range between 869 and 4723 (mean = 2578.7, n = 6) which is similar the range seen at Zebra River, from 724 to 3098 ppm (mean = 1258 ppm, n = 23). Mg/Ca at Driedoornvlakte is 0.005 to 0.002 (mean = 0.008, n = 6), and Zebra River shows values of 0.012 to 0.021 (mean = 0.016, n = 23). Sediment Mg/Ca content varies between Driedoornvlakte and Zebra River with values ranging between 0.007 and 0.010 (mean = 0.009, n = 5) and 0.007 to 0.023 (mean = 0.014, n = 9), respectively. Sr concentrations have similar ranges with 796 to 1363 ppm (mean = 987, n = 9) at Zebra River, and 755 to 1637 ppm (1082 ppm, n = 5) at Driedoornvlakte.

|  | **Driedoornvlakte** | **Zebra River** | **Omkyk** | **Zwartmodder** |
| --- | --- | --- | --- | --- |
| **Latitude** | 23° 51' 36.83'' S | 24° 30' 49.38'' S | 24°48'19.00"S | 24°53'41.00"S |
| **Longitude** | 16° 39' 50.57'' E | 16° 12'13.24'' E | 16°13'45.00"E | 16°19'31.00"E |
| **Setting** | Mid-ramp, reef-derived *Cloudina*, not in in-situ | Proximal mid-ramp, in-situ attached to elongate stromatolites/thrombolites and inter-reefal detritus | Uppermost mid-ramp to distal, inner ramp, algal mat community mainly in-situ, possibly lagoonal | Proximal, inner ramp, not in-situ, possibly lagoonal |
| **Data source** | Bedding planes and within beds | Bedding planes, inter-reef debris between elongate stromatolites/thrombolites | Bedding planes, associated with microbial mats | Bedding planes |
| **Dominant lithology** | Grainstone | Wackestone-packstone | Wackestone-packstone | Wackestone |
| **Relative water depth** | Subtidal, above wave base | Subtidal, above wave base | Shallow subtidal | Very shallow subtidal |
| **Relative hydrodynamic energy** | High | Medium | Low | Very-low |
| **Dominant early diagenetic fabric and regime** | Large (< 100 mm) syn-sedimentary pseudomorphed aragonitic botryoids. Marine phreatic, reefal. | Syn-sedimentary pseudomorphed aragonitic botryoids, < 10 mm.  Marine phreatic, reefal. | Dissolution of Aragonite/High-Mg calcite. Meteoric phreatic. | Dissolution of Aragonite/High-Mg calcite. Meteoric phreatic. |

**Table S1:** Summary of depositional and early diagenetic regime, and *Cloudina* data sources, for four coeval localities from the Upper Omkyk Member, Nama Group, Namibia.

**Table S2:** Summary of *Cloudina* characteristics from four contemporary localities, Upper Omkyk Member, Zaris Sub-basin, Namibia.

| *Cloudina* | Localities | | | |
| --- | --- | --- | --- | --- |
|  | Driedoornvlakte | Zebra River | Omkyk | Zwartmodder |
| Laminae | X | X |  | ? |
| Paired Laminae | X | X |  | ? |
| Inter-lamina Cement | X | X |  | ? |
| Aragonite Needle Inter-laminae Cement | X |  |  |  |
| Inter-cloudinomorph Cement | X |  |  |  |
| Intra-lamina Cement | X | X |  |  |
| Brittle Deformation | X | X |  |  |
| Ductile Deformation |  | X |  |  |
| Dissolution |  |  | X | X |
| Average Sinuosity | 1.04 | 1.21 | 1.07 | 1.04 |
| Mean Lamina Thickness | 5 µm | 6.5 µm |  |  |
| Mean Paired Laminae Thickness | 30 µm | 19.3 µm |  | 15.4 µm |
| Mean Maximum Wall Thickness:Tube Width | 0.235 | 0.213 |  | 0.190 |

**Table S3**: combined Sr concentration (ppm) data collected *Cloudina*-associated cements, pseudomorphed aragonitic botryoids and sediment for Driedoornvlakte.

| Driedoornvlakte *Cloudina*-associated cement Sr concentrations (ppm) | | | | | | | | | |
| --- | --- | --- | --- | --- | --- | --- | --- | --- | --- |
|  | Lamina | Inter-laminae cement | Inter-funnel Cement | Cloudina Wall | Mutual Cement | Inter-cloudinomorph | Intra-cloudinomorph | Botryoids | Sediment |
| Mean | 1199 | 1566 | 869 | 1119 | 1212 | 1409 | 1798 | 2579 | 1082 |
| Min | 777 | 1221 | 457 | 189 | 393 | 521 | 195 | 869 | 755 |
| Max | 1617 | 1804 | 1747 | 2773 | 1958 | 3003 | 3267 | 4723 | 1637 |
| n | 3 | 3 | 16 | 13 | 5 | 9 | 8 | 6 | 5 |

**Table S4**: Sr concentration (ppm) data collected *Cloudina*-associated cements, pseudomorphed aragonitic botryoids and sediment for Zebra River.

| Zebra River *Cloudina*-associated cement Sr concentration (ppm) | | | | | |
| --- | --- | --- | --- | --- | --- |
|  | Lamina | Inter-lamina Cement | Inter-funnel | Botryoid | Sediment |
| Mean | 1064 | 1490 | 1169 | 1258 | 987 |
| Min | 719 | 819 | 904 | 724 | 796 |
| Max | 1596 | 2064 | 1567 | 3098 | 1363 |
| n | 7 | 12 | 15 | 23 | 9 |

**Table S5**: Mg/Ca ratio of *Cloudina*-associated cements, pseudomorphed aragonitic botryoids and sediment for Zebra River.

| Zebra River *Cloudina*-associated cement Mg/Ca ratio | | | | | |
| --- | --- | --- | --- | --- | --- |
|  | Lamina | Inter-lamina Cement | Inter-funnel | Botryoid | Sediment |
| Mean | 0.015 | 0.017 | 0.017 | 0.016 | 0.014 |
| Min | 0.010 | 0.011 | 0.013 | 0.012 | 0.007 |
| Max | 0.020 | 0.025 | 0.023 | 0.021 | 0.023 |
| n | 7 | 12 | 15 | 23 | 9 |

**Table S6**: Mg/Ca ratio of *Cloudina*-associated cements, pseudomorphed aragonitic botryoids and sediment for Driedoornvlakte.

| Driedoornvlakte *Cloudina*-associated cement Mg/Ca ratio | | | | | | | | | |
| --- | --- | --- | --- | --- | --- | --- | --- | --- | --- |
|  | Laminae | Inter-lamina | Inter-Funnel | *Cloudina* Wall | Mutual Cement | Inter-cloudinomorph | Intra-cloudinomorph | Botryoids | Sediment |
| Mean | 0.128 | 0.009 | 0.007 | 0.216 | 0.229 | 0.015 | 0.010 | 0.008 | 0.009 |
| Min | 0.007 | 0.008 | 0.004 | 0.005 | 0.007 | 0.006 | 0.008 | 0.005 | 0.007 |
| Max | 0.367 | 0.012 | 0.012 | 0.553 | 0.621 | 0.031 | 0.016 | 0.015 | 0.010 |
| n | 3 | 3 | 16 | 13 | 3 | 9 | 8 | 6 | 5 |

**Table S7**: p-values of *Cloudina*-associated cements, pseudomorphed aragonitic botryoids and sediment Sr concentration, Zebra River. Yellow p-values highlight significant values.

| Zebra River *Cloudina*-associated cements T-test p-values (Sr concentration) | | | | | |
| --- | --- | --- | --- | --- | --- |
|  | Lamina | Inter-lamina | Inter-funnel | Botryoids | Sediment |
| Laminae | X | 0.016 | 0.406 | 0.450 | 0.506 |
| Inter-lamina | X | X | 0.014 | 0.180 | 0.001 |
| Inter-funnel | X | X | X | 0.548 | 0.030 |
| Botryoids | X | X | X | X | 0.073 |
| Sediment | X | X | X | X | X |

| Zebra River *Cloudina*-associated cements T-test p-value (Mg/Ca) | | | | | |
| --- | --- | --- | --- | --- | --- |
|  | Laminae | Inter-lamina | Inter-funnel | Botryoids | Sediment |
| Laminae | X | 0.294 | 0.156 | 0.419 | 0.674 |
| Inter-lamina | X | X | 0.921 | 0.372 | 0.123 |
| Inter-funnel | X | X | X | 0.213 | 0.047 |
| Botryoids | X | X | X | X | 0.129 |
| Sediment | X | X | X | X | X |

**Table S8**: p-values of *Cloudina*-associated cements, pseudomorphed aragonitic botryoids and sediment Mg/Ca for Zebra River. Yellow p-values highlight significant values.

**Table S9**: p-values of *Cloudina riemkeae*-associated cements and the inclusion-rich spar strontium concentration (ppm) for Driedoornvlakte. Yellow p-values highlight significant values.

| Driedoornvlakte *Cloudina riemkeae*-associated cements T-test p-values (Sr concentration) | | | | | |
| --- | --- | --- | --- | --- | --- |
|  | Lamina | Inter-lamina | Inter-funnel | *Cloudina* Wall | IR Spar |
| Lamina | X | 0.623 | 0.256 | 0.136 | 0.201 |
| Inter-Laminae | X | X | 0.041 | 0.009 | 0.023 |
| Inter-funnel Cement | X | X | X | 0.002 | 0.291 |
| *Cloudina* Wall | X | X | X | X | 0.011 |
| IR Spar | X | X | X | X | X |

**Table S10**: p-values of *Cloudina hartmannae*-associated cements, pseudomorphed aragonitic botryoids and sediment strontium concentration (ppm) for Driedoornvlakte. Yellow p-values highlight significant values.

| Driedoornvlakte *Cloudina hartmannae*-associated cements T-test p-values (Sr concentration) | | | | | | |
| --- | --- | --- | --- | --- | --- | --- |
|  | *Cloudina* Wall | Mutual Cement | Inter-cloudinomorph | Intra-cloudinomorph | Botryoids | Sediment |
| *Cloudina* Wall | X | 0.206 | 0.376 | 0.937 | 0.130 | 0.062 |
| Mutual Cement | X | X | 0.667 | 0.324 | 0.065 | 0.732 |
| Inter-Cloudinomorph | X | X | X | 0.424 | 0.043 | 0.422 |
| Intra-Cloudinomorph | X | X | X | X | 0.197 | 0.196 |
| Botryoids | X | X | X | X | X | 0.035 |
| Sediment | X | X | X | X | X | X |

**Table S11**: p-values of *Cloudina riemkeae*-associated cements Mg/Ca ratio for Driedoornvlakte. Yellow p-values highlight significant values.

| Driedoornvlakte *Cloudina riemkeae*-associated cements T-test p-values (Mg/Ca) | | | | | |
| --- | --- | --- | --- | --- | --- |
|  | Lamina | Inter-lamina | Inter-funnel | *Cloudina* Wall | Inclusion-rich Spar |
| Lamina | X | 0.473 | 0.444 | 0.026 | 0.714 |
| Inter-lamina | X | X | 0.061 | 0.008 | 0.733 |
| Inter-funnel | X | X | X | 1.281E-08 | 0.179 |
| *Cloudina* Wall | X | X | X | X | 1.29E-04 |
| Inclusion-rich Spar | X | X | X | X | X |

**Table S12**: p-values of *Cloudina hartmannae*-associated cements, pseudomorphed aragonitic botryoids and sediment Mg/Ca ratio for Driedoornvlakte. Yellow p-values highlight significant values.

| Driedoornvlakte *Cloudina hartmannae*-associated cements T-test p-values (Mg/Ca) | | | | | | | |
| --- | --- | --- | --- | --- | --- | --- | --- |
|  | Lamina | Mutual Cement | *Cloudina* Wall | Inter-cloudinomorph | Intra-Cloudinomorph | Botryoids | Sediment |
| Lamina | X | 0.703 | N/A | N/A | 0.062 | N/A | N/A |
| Mutual Cement | X | X | 0.084 | 0.051 | 0.062 | 0.107 | 0.146 |
| *Cloudina* Wall | X | X | X | 0.289 | 0.691 | 0.307 | 0.419 |
| Inter-cloudinomorph | X | X | X | X | 0.131 | 0.078 | 0.125 |
| Intra-Cloudinomorph | X | X | X | X | X | 0.305 | 0.427 |
| Botryoids | X | X | X | X | X | X | 0.693 |
| Sediment | X | X | X | X | X | X | X |


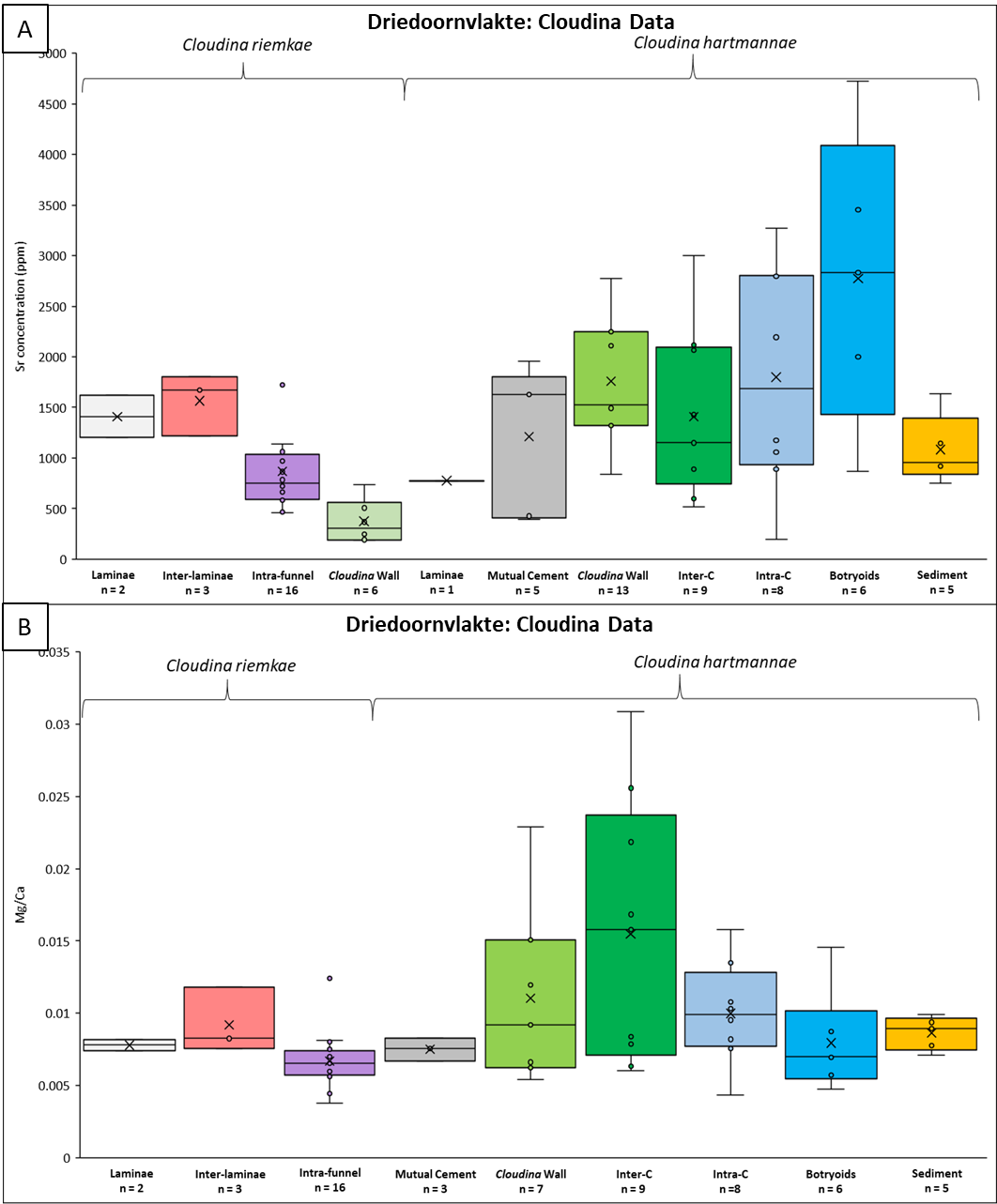


**Figure S1**: EMPA data of *Cloudina*-associated cement collected at Driedoornvlakte, separated by *Cloudina* species. **A**: Sr concentration (ppm). **B:** Mg/Ca ratio.

**References**

1 Grotzinger, J. P., Watters, W. A. & Knoll, A. H. Calcified metazoans in thrombolite-stromatolite reefs of the terminal Proterozoic Nama Group, Namibia. *Palaeobiology* **26**, 334-359 (2000).

2 Penny, A. M. *et al.* Ediacaran metazoan reefs from the Nama Group, Namibia. *Science* **344**, 1504-1506 (2014).

3 Johnson, J. & Grotzinger, J. Affect of sedimentation on stromatolite reef growth and morphology, Ediacaran Omkyk Member (Nama Group), Namibia. *South African Journal of Geology,* **109**, 87-96 (2006).

4 Wood, R. *et al.* Dynamic redox conditions control late Ediacaran metazoan ecosystems in the Nama Group, Namibia. *Precambrian Research* **261**, 252-271 (2015).

5 Wood, R. *et al.* Flexible and responsive growth strategy of the Ediacaran skeletal Cloudina from the Nama Group, Namibia. *Geology* **45**, 259-262 (2017).

6 Shore, A., Wood, R., Curtis, A. & Bowyer, F. Multiple branching and attachment structures in cloudinomorphs, Nama Group, Namibia. *Geology*, doi:10.1130/G47447.1 (2020).
